# Ecological Niche Modeling Reveals Historical Population Dynamics and Future Climate Response of the Carnivorous Plant *Nepenthes mirabilis* in Southeast Asia

**DOI:** 10.1002/ece3.72707

**Published:** 2025-12-16

**Authors:** Zhilong Huang, Wenya Yu, Wenjun Lv, Chuxin Liang, Rongjing Zhang, Archie Along, Hanghui Kong, Wei Gong

**Affiliations:** ^1^ College of Life Sciences South China Agricultural University Guangzhou China; ^2^ College of Ecology and Environment Nanjing Forestry University Nanjing China; ^3^ Department of Biology Caraga State University Butuan City Philippines; ^4^ Key Laboratory of National Forestry and Grassland Administration on Plant Conservation and Utilization in Southern China, South China Botanical Garden Chinese Academy of Sciences Guangzhou China

**Keywords:** climate change, dynamic history, ecological niche modeling, evolutionary inference, *Nepenthes mirabilis*, suitable distribution range

## Abstract

Climate change and environmental factors are reshaping natural habitats, threatening species diversity and genetic resources. *Nepenthes mirabilis* , a dioecious carnivorous plant and vulnerable species, is mainly distributed in Southeast Asia, one of the key biodiversity hotspots and conservation priority areas. In this study, we used the MaxEnt model to predict the potential distribution of *N. mirabilis* across past, present, and future climate scenarios, based on 513 occurrence records and five key environmental variables of Annual Mean Temperature, Temperature Seasonality, Annual Precipitation, April Precipitation, and December Precipitation. The model demonstrated excellent predictive performance with high AUC and TSS values. Annual Precipitation was identified to be the most important climate factor influencing the geographical distribution of *N. mirabilis* . Wallacea and New Guinea are suggested as likely centers of the geographical origin of *N. mirabilis* , from where it expanded to the Sundaic region through the land bridge during glacial periods and subsequently colonized the Philippines and Indochina. Under future scenarios, the most suitable climate scenario for the development of *N. mirabilis* is the Sustainable Development Pathway. As temperature rises, suitable habitats are projected to shift toward higher latitudes, with low‐latitude populations facing increasing risks. By integrating Ecological Niche Modeling with evolutionary inference, the study interprets how historical and future climate oscillations shape geographical distributions in Southeast Asia and provides in situ and *ex situ* conservation strategies for *N. mirabilis.* These findings also contribute to a broad understanding of species evolution and population dynamics across subtropical and tropical biodiversity hotspots.

## Graphical abstract

The carnivorous pitcher plant *Nepenthes mirabilis* likely originated in Wallacea and New Guinea, and expanded northward into the Sundaic, Indochina, and Philippine regions during glacial and interglacial periods. In response to future climate change, the predicted geographical distributions are projected to a latitudinal shift toward higher latitudes with high‐suitability habitats, contracting in low‐latitude regions due to high greenhouse gas emission scenarios.

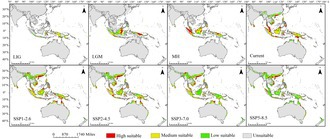

## 1 Introduction

Climate change is a critical driver of global biodiversity loss (Wan et al. 2021). As greenhouse gas emissions increase, global temperatures continue to rise (Folland et al. 2001; IPCC 2013). Projections indicate that the global average temperature will rise by 3.6°C–5°C by 2100 (IPCC 2013; Pacifici et al. 2015). This ongoing warming poses severe threats to ecosystems, species survival, and biodiversity worldwide (Williams et al. 2003; Thomas et al. 2004; Raxworthy et al. 2008), compelling organisms to adapt to rapidly changing climatic conditions (Auer and King 2014). Numerous studies have demonstrated that many plant species are shifting their distribution toward higher latitudes or elevations in response to rising temperature (Hickling et al. 2006; Chen et al. 2011; He et al. 2019). These shifts highlight the need to understand how plant distributions respond to climate change, both spatially and temporally (Khan et al. 2022; Zhao et al. 2024). As we know, paleo‐climate data can be used to model historical climate suitability, offering insights into species' evolutionary trajectories and potential past migration routes (Carnaval and Moritz 2008; Park et al. 2022). Similarly, projections based on future climate scenarios can also help to predict potential distribution changes and identify areas at risk (Wang et al. 2024). By examining plant responses to past, present, and future climatic conditions, conservation strategies can be better informed, thereby helping to mitigate the adverse effects of climate change on biodiversity (Merow and Silander 2014; Wang et al. 2024).

Southeast Asia is located at the intersection of the Eurasian, Indo‐Australian, and Pacific‐Philippines tectonic plates (Hall 2013; Ma and Song 2023). The region comprises four biogeographic subregions: the Indochina, Sundaic, Philippines, and Wallacea (Woodruff 2010; Ma and Song 2023). Additionally, New Guinea is considered part of Southeast Asia's geographical scope (Ma and Song 2023). Since the early Eocene (~50 million years ago), global climate changes have caused significant alterations in Southeast Asia's environment. Periodic fluctuations in sea levels during the Pliocene and Pleistocene caused the continuous merging and isolation of Southeast Asia's islands with Sundaland (Meijaard 2004; Hall 2009; Janssens et al. 2016). Despite these geological changes, Southeast Asia has maintained a relatively stable tropical climate, providing favorable environmental conditions for plant growth and reproduction (Mittermeier et al. 1999). Specifically, the lowering of sea levels during glacial periods enabled land bridges to form, facilitating plant migrations and gene flow, whereas interglacial sea‐level rises led to island isolation, promoting speciation and adaptive evolution (Audley‐Charles 1983; De Bruyn et al. 2014; Roalson and Roberts 2016). Today, Southeast Asia is characterized by a complex geographic landscape comprising over 20,000 islands (Lohman et al. 2011; Van Welzen et al. 2011). These unique and complex geological and climatic processes have profoundly played a key role in generating and maintaining the region's exceptional floral biodiversity (Zhu 2013; Buerki et al. 2014).

Ecological niche modeling (ENM) is a widely used method in ecology and biogeography to predict the potential distribution of species based on environmental variables. It has become an essential tool for understanding species‐environment relationships and spatial biodiversity patterns. Projecting species distributions under past climatic scenarios based on palaeoclimatic data is a widely used approach in ENM and historical biogeography studies. As is known, ENM based on paleoclimatic data can reveal species' evolutionary history, inferring historical range dynamics, identifying potential refugia, and understanding how climatic fluctuations have shaped species' distribution patterns over time, thus offering insights into historical distribution shifts and the evolutionary development of vegetation types (Park et al. 2022). Moreover, reconstructing past geographic distributions is not only valuable for understanding a species' evolutionary and biogeographic history, but it can also provide important context for predicting future responses to climate change. By comparing historical range shifts with projected future changes, we can gain deeper insights into the species' climatic tolerances, dispersal capabilities, and potential adaptive responses, thereby improving the robustness and ecological relevance of our predictions. In contrast, models based on future climate scenarios can provide predictions on how species respond to habitat changes, helping to inform ecological management strategies (Sinclair et al. 2010). Currently, ENM is commonly employed to simulate species distribution across past, present, and future periods, serving as a powerful framework for both evolutionary and conservation‐oriented research (Di Febbraro et al. 2016; Wang et al. 2024).

The maximum entropy (MaxEnt) algorithm (Phillips et al. 2006) is a machine learning model that integrates environmental variables and species distribution data to predict suitable distribution areas for a species based on the principle of maximum entropy (Mahatara et al. 2021). It also characterizes the relationship between species distribution and environmental variables (Merow and Silander 2014; Ngarega et al. 2022). Among the presence‐only modeling approaches, MaxEnt shows comparatively better prediction performance for making predictions from limited species distribution data (Phillips et al. 2006; Phillips and Dudík 2008; Elith et al. 2011; Merow et al. 2013; Nair and Peterson 2023; Zhou et al. 2023). Owing to its high accuracy and reliability, MaxEnt was chosen to characterize the geographical distribution of *Nepenthes mirabilis* for the past, present, and future in the current study.

*Nepenthes mirabilis* belongs to the *Nepenthes* genus within the Nepenthaceae family (Jebb and Cheek 1997; Robinson et al. 2009). *Nepenthes* species are dioecious carnivorous plants, with approximately 160 to 180 species worldwide (Ellison and Adamec 2018). The pitcher plant trap is a striking example of convergent evolution, as is seen in the unrelated lineages of pitcher plants (especially the families Nepenthaceae, Sarraceniaceae, and Cephalotaceae), which independently evolved but remarkably have similar traps as adaptations to survival in nutrient‐poor environments (Thorogood et al. 2018). *Nepenthes* represents one of the ca. 6% of flowering plant genera that are dioecious and is considered to be the only dioecious genus among carnivorous plants (Renner 2014; Saul et al. 2023). *Nepenthes mirabilis* is native to the tropical regions of Southeast Asia and Australia (Cheek and Jebb 2001). Due to the combined impacts of climate change and human activities, the population of *N. mirabilis* has been rapidly declining. In 2014, it was listed as a vulnerable species on the IUCN Red List of Threatened Species (Clarke 2014).

The unique morphology, carnivorous nature, and dioecious reproductive system of *N. mirabilis* contribute to its high scientific value (Clarke et al. 2017), which makes it an excellent model for studying plant adaptation and survival under changing climatic conditions. Previous studies have revealed its phylogenetic position (Zhu et al. 2018; Yao et al. 2019) and carnivory mechanisms (Schulze et al. 1999; Moran et al. 1999). However, the origin, historical evolutionary characteristics, and migration routes of *N. mirabilis* remain unclear. While Schwallier et al. (2016) indeed conducted ecological niche modeling (ENM) across multiple *Nepenthes* species and provided valuable insights into the genus‐level distribution, their analysis primarily focused on Southeast Asia as a region and did not specifically address *N. mirabilis* at the species level. In particular, their study did not explore the ecological niche dynamics and distribution patterns of *N. mirabilis* within China, which is a significant part of this species' range and an area where unique historical and future patterns may occur. Additionally, this study focused primarily on the current distribution and future habitat projections, without addressing the potential impacts of past climatic conditions. The current study utilizes the MaxEnt model to analyze the potential suitable distribution of a single widespread species, *N. mirabilis* , under paleo‐climatic, current, and future climate conditions. We collected global distribution data for *N. mirabilis* and combined it with paleo‐climatic data (Last Interglacial: LIG; Last Glacial Maximum: LGM; and Mid‐Holocene: MH) along with current climate data to explore the dynamic historical migration of *N. mirabilis* . Additionally, we predicted the suitable distribution areas of *N. mirabilis* under various future climate scenarios for the 2080s–2090s, including SSP1‐2.6, SSP2‐4.5, SSP3‐7.0, and SSP5‐8.5. This study sheds light on the dynamic historical evolution of *N. mirabilis* , offering a new perspective on the evolutionary history of Southeast Asian vegetation and providing novel insights into conservation strategies for *N. mirabilis* populations.

## 2 Materials and Methods

### 2.1 Species Distribution Data

**FIGURE 1:**
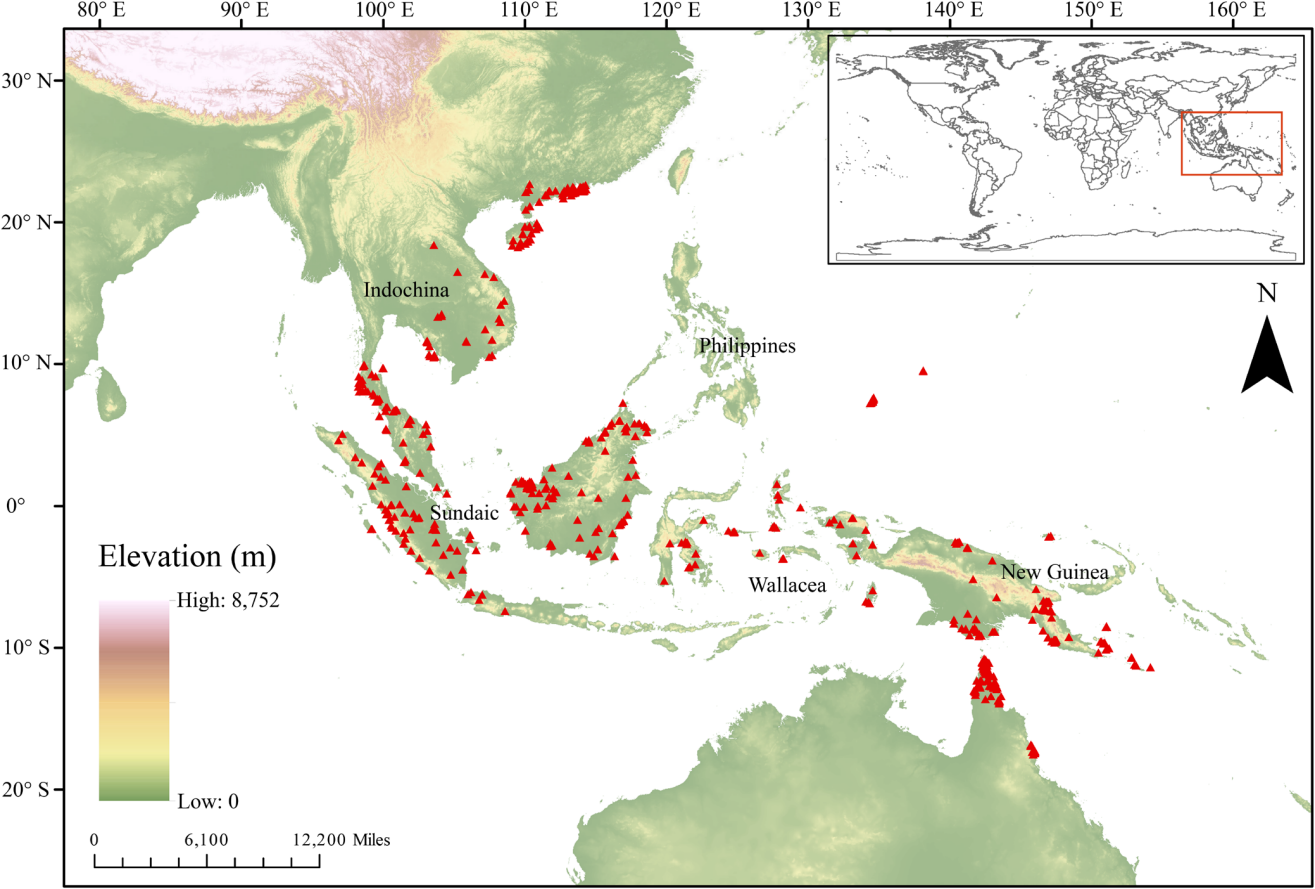
Distribution map of *Nepenthes mirabilis* records used in the modeling analysis. Red triangles represent species occurrence points.

We gathered species distribution data for *Nepenthes mirabilis* from the Global Biodiversity Information Facility (GBIF, https://www.gbif.org/; accessed 6 March 2024), the China Virtual Herbarium (CVH, https://www.cvh.ac.cn/; accessed 6 March 2024), and field survey sampling locations. The data primarily cover the distribution of *N. mirabilis* in Southeast Asia. For some distribution points lacking latitude and longitude information, we used the Geo Online website (https://map.jiqrxx.com/; accessed 6 March 2024) to convert these points into more precise coordinates. Duplicate records with identical coordinates were removed to avoid sampling bias. Spatial autocorrelation was minimized using a thinning approach, by means of R package “spThin” v.0.1.0 function applied at 5 km × 5 km grid resolution, ensuring only one occurrence record per grid (Aiello Lammens et al. 2015). This threshold was chosen to match the ~5 km spatial resolution of the environmental variables and to minimize overrepresentation of densely sampled localities. Finally, a total of 513 occurrence records were acquired for modeling (Figure 1).

### 2.2 Environmental Variables

We downloaded 68 environmental variables, including 2.5 arcminute bioclimatic variables, monthly average maximum temperature, monthly average minimum temperature, monthly average precipitation, and elevation, from the WorldClim 2.1 climate data (https://worldclim.org/; accessed 6 March 2024) for the period 1970–2000 to represent the current environmental conditions. Additionally, we obtained slope and aspect data with a resolution of 30 arcseconds from the FAO soil portal (https://www.fao.org/; accessed 6 March 2024) and resampled them to 2.5 arcminutes using ArcGIS v.10.8.

We downloaded paleoclimate data from WorldClim 1.4, based on the Community Climate System Model version 4 (CCSM4; Gent et al. 2011), covering the Mid‐Holocene (MH; ~6 ka), the Last Glacial Maximum (LGM; ~22 ka), and the Last Interglacial (LIG; ~125 ka). The MH and LGM data were downloaded at a spatial resolution of 2.5 arcminutes, while the LIG data were obtained at a resolution of 30 arcseconds and subsequently resampled to 2.5 arcminutes using ArcGIS v10.8. Future climate data were sourced from WorldClim 2.1 using the Beijing Climate Center Climate System Model (BCC‐CSM2‐MR), with four Shared Socioeconomic Pathways (SSPs): SSP1‐2.6 (Sustainable Development), SSP2‐4.5 (Moderate Development), SSP3‐7.0 (Partial Development), and SSP5‐8.5 (General Development). We selected the 2.5 arcminute resolution environmental variables for the 2090s period (2080s–2100s) for all four SSP scenarios.

### 2.3 Key Environmental Variable Selection

Multicollinearity among environmental variables can lead to overfitting in model predictions (Graham 2003). Therefore, we selected key environmental variables based on the initial variable contributions and importance rankings from the MaxEnt model output (Table S1). We identified key environmental variables using Variance Inflation Factor (VIF) and Pearson Correlation Analysis (Pearson 2010; Sillero 2011), ensuring no multicollinearity and detecting linear correlations among variables. Variables with VIF ≥ 2.5 or Pearson correlation coefficient |*r*| ≥ 0.7 were considered highly correlated and excluded from further analysis. The VIF was calculated using the vif_func function from the “fmsb” R package and the lm() function from the “MASS” package, whereas Pearson Correlation Analysis was conducted using the “corrplot” R package. Based on these criteria, several variables such as bio05 (Max Temperature of Warmest Month), bio06 (Min Temperature of Coldest Month), and bio15 (Precipitation Seasonality) were removed due to high multicollinearity. The final five key environmental variables selected for modeling were bio01 (Annual Mean Temperature), bio04 (Temperature Seasonality), bio12 (Annual Precipitation), prec04 (April Precipitation), and prec12 (December Precipitation). All retained variables showed Pearson |*r*| < 0.7 and VIF < 2.5, indicating low multicollinearity and suitability for ecological niche modeling (Figure S1).

### 2.4 Model Parameter Optimization

When constructing the MaxEnt model, it is necessary to set the Regularization Multiplier (RM) and Feature Combination (FC) parameters, and select from the features provided by the model, including Linear (L), Quadratic (Q), Hinge (H), Threshold (T), and Product (P) features. The choice of feature combinations and the Regularization Multiplier directly affects model complexity, and excessive complexity may lead to overfitting (Radosavljevic and Anderson 2014). We set RM values ranging from 1 to 4 and tested four feature combinations (LQ, LQH, LQHP, and LQHPT), resulting in 16 parameter combinations (Table S2). We used the R package “ENMeval” (Muscarella et al. 2014) to evaluate model performance by calculating the Delta Akaike Information Criterion corrected (Delta.AICc), the difference between training and testing AUC (AUC.DIFF), and the 10% Training Omission Rate (OR10) (Figure S2; Zhao et al. 2021; Yang et al. 2022). Among these models, the parameter set with RM = 1 and the LQHPT feature combination was ultimately selected because it achieved the lowest AICc value, indicating the best balance between model fit and complexity, while also maintaining low omission rates and minimal overfitting (lowest AUC.DIFF). This combination was therefore considered to provide the most parsimonious and robust model for predicting the potential distribution of *N. mirabilis* .

### 2.5 Model Construction and Accuracy Evaluation

Species occurrence data and current environmental variables were imported into the MaxEnt software (v.3.4.4) (Elith et al. 2011; Phillips and Dudík 2008), with the feature combination LQHPT. The options “Create response curves,” “Generate prediction maps,” and “Perform jackknife to measure variable importance” were selected, with the output format set to Logistic and the output file type set to asc. The Regularization Multiplier was set to 1, with 75% of occurrence records randomly selected for training and 25% for testing. The model was run for 5000 iterations with 20 repetitions, and the results were averaged. For the projection layers, the folder containing the paleoclimate or future climate environmental variable files was selected to construct the ecological niche models for past and future climates.

We used two methods to evaluate the model's accuracy: the Area Under the Curve (AUC) of the Receiver Operating Characteristic (ROC) and the True Skill Statistics (TSS) (Liang et al. 2018; Merow et al. 2013). The AUC ranges from 0 to 1, with values 0.9–1 indicating excellent and highly accurate predictions (Hosni et al. 2020). TSS evaluates the overall prediction accuracy of the model by comparing the true positive rate and the false positive rate. TSS ranges from −1 to +1, with values greater than 0.75 indicating an excellent model (Hosni et al. 2020; Kong et al. 2021).

### 2.6 Model Output and Data Analysis

The model outputs suitability probability values (*p* values), which range from 0 to 1. A *p* value of 0 indicates unsuitable areas, and a *p* value of 1 indicates the most suitable areas. The average result from 20 repetitions, saved as the _avg.asc file, is used as the model's prediction output and imported into ArcGIS v.10.8 for conversion into raster format. The reclassification tool is used to divide the suitability levels into four categories: 0–0.1 for unsuitable areas, 0.1–0.3 for low suitability, 0.3–0.5 for moderately suitable, and 0.5–1 for highly suitable areas (Liu et al. 2021; Wu et al. 2022). The suitability distribution areas and centers of *N. mirabilis* under current, past, and future climate scenarios are analyzed using ArcGIS v.10.8 and SDMtoolbox (Brown 2014). Changes in the area and range of suitability distribution across different periods are compared, and the expansion and contraction of suitable distribution areas of *N. mirabilis* are analyzed, thereby inferring the spatiotemporal dynamics of its suitable distribution from past to present and future climates.

## 3 Result

### 3.1 Model Performance and Key Environmental Variables

**FIGURE 2:**
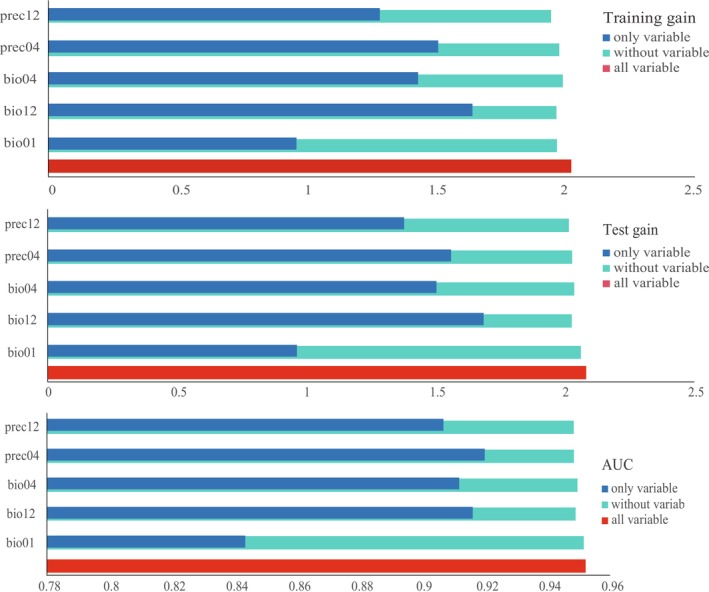
Jackknife test results of the potential habitat suitability model for *Nepenthes mirabilis* . “Only variable” indicates the model performance when using each environmental variable individually; “Without variable” indicates the model performance when excluding the specified variable; “All variables” indicates the model incorporating all environmental variables.

The AUC and TSS values from the 20 repeated runs of the MaxEnt model under the current climate are both greater than 0.93 (Table S3). Furthermore, the average AUC and TSS values are both 0.95 (Table S3). These results indicate that the distribution model for *N. mirabilis* is highly accurate. The results from the Jackknife test and the contribution of five environmental variables to the MaxEnt model reveal the relative importance of these variables in shaping the distribution of *N. mirabilis* (Figure 2; Table 1).

**TABLE 1:** Contribution and importance of the five variables to the MaxEnt model for *Nepenthes mirabilis*.

Variable	Contribution	Logistic > 0.5	Logistic max	Temperature/Precipitation
bio01	8.8	22.11–27.99	26.22	T (°C)
bio04	10.5	33.93–523.59	221.55	T (−)
bio12	54.7	1491.36–4132.68	1656.96	P (mm)
prec04	18.7	102.22–630.30	573.92	P (mm)
prec12	7.3	168.92–775.50	706.13	P (mm)

*Note:* Logistic > 0.5 indicates the range of the variable where the predicted suitability exceeds 0.5. Logistic max represents the value at which the model predicts the maximum habitat suitability for each variable.

**FIGURE 3:**
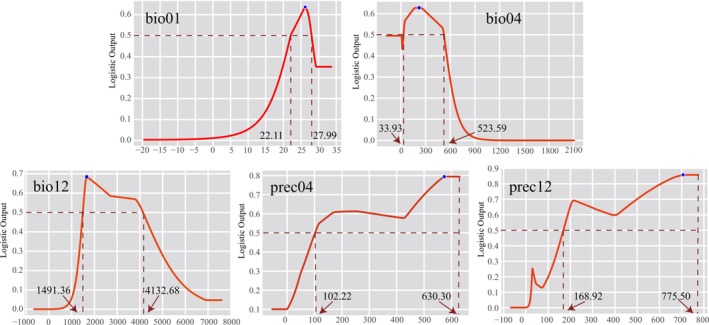
Response curves of key environmental variables influencing the potential suitable distribution of *Nepenthes mirabilis* . The horizontal red dashed line indicates the threshold of high habitat suitability (logistic output > 0.5). The vertical red dashed lines represent the range of variable values corresponding to the highest predicted suitability. The blue solid dot denotes the point with the highest predicted suitability.

All five environmental variables demonstrated high contributions and importance in the model, highlighting their critical roles in the MaxEnt predictions (Figure 2; Table 1). Among them, Annual Precipitation (bio12) made the largest contribution, accounting for 54.7% of the model's predictive performance, followed by April Precipitation (prec04, 18.7%) and Temperature Seasonality (bio04, 10.5%). The response curves for the five environmental variables indicate that *N. mirabilis* is most likely to occur in environments with an Annual Mean Temperature (bio01) between 22.11°C and 27.99°C, a temperature fluctuation (bio04) ranging from 0.34°C to 5.24°C, and Annual Precipitation (bio12) between 1491 and 4133 mm (Figure 3). Among them, a lower bio04 value suggests that *N. mirabilis* tends to occur in areas with more stable temperatures.

### 3.2 Potential Distribution Areas, Dynamics of Suitable Habitats, and Vegetation Changes Under Past and Current Climates

**FIGURE 4:**
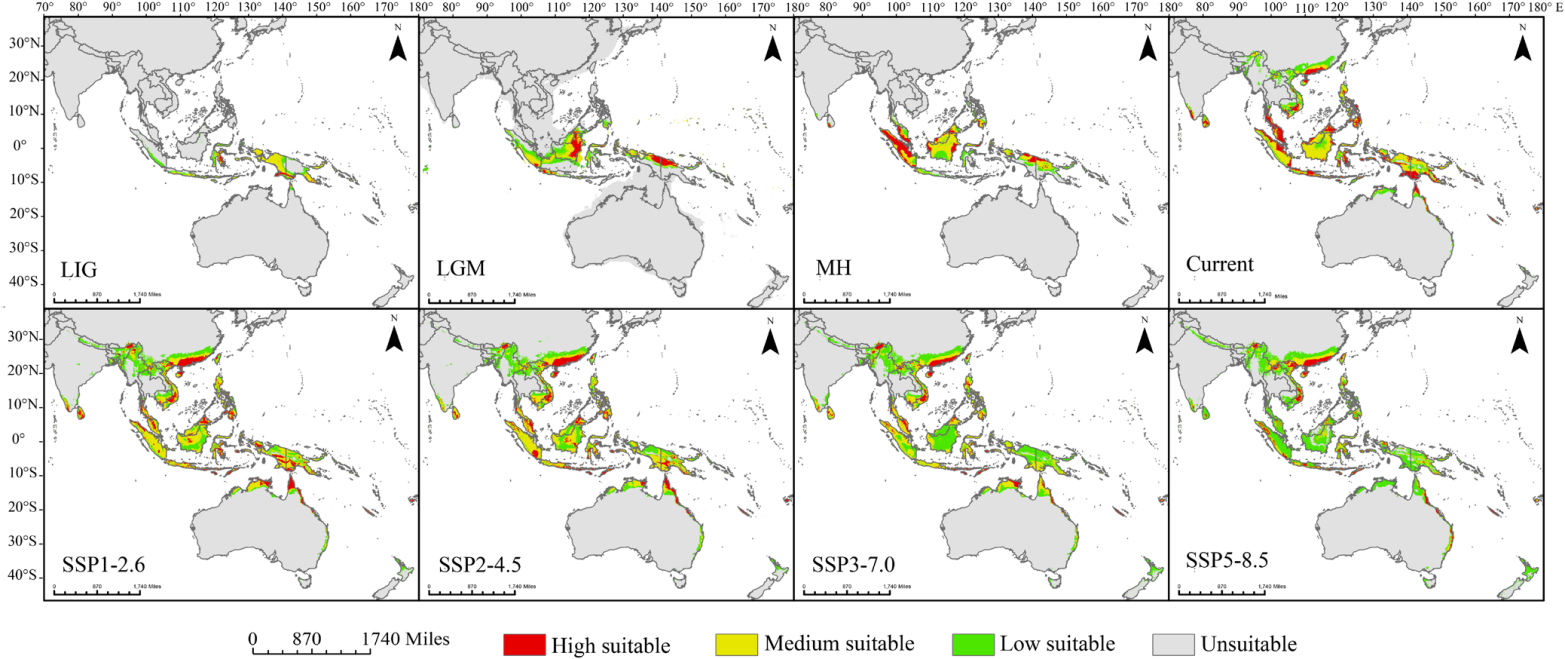
Predicted potential suitable distribution of *Nepenthes mirabilis* under paleoclimatic, current, and future climate scenarios. Red areas indicate highly suitable habitats (logistic probability > 0.5); yellow areas indicate moderately suitable habitats (0.3 < logistic probability ≤ 0.5); green areas indicate low suitability (0.1 < logistic probability ≤ 0.3); and gray areas represent unsuitable habitats (logistic probability ≤ 0.1).

The potential suitable distribution areas of *N. mirabilis* during the LIG, LGM, MH, and present periods were 92.67 × 10^4^ km^2^, 197.13 × 10^4^ km^2^, 201.59 × 10^4^ km^2^, and 410.77 × 10^4^ km^2^, respectively (Table 2). Over time, the suitable distribution areas of *N. mirabilis* have undergone substantial changes (Figure 4; Tables 2 and 3; Video S1).

**TABLE 2:** The area of suitable distribution range in each period, as well as the area of expansion and contraction of the suitable range between two periods for *N. mirabilis*.

Period	Total	Low	Medium	High		Expansion	Contraction	Stabilization
	×10^4^ km^2^					×10^4^ km^2^		
LIG	92.67	30.49	54.79	7.39	LIG‐LGM	89.63	48.76	43.50
LGM	197.13	78.70	87.75	30.67	LGM‐MH	62.44	57.98	138.16
MH	201.59	50.96	96.51	54.12	MH‐Current	209.98	3	193.49
Current	410.77	108.91	176.02	125.84				
SSP1‐2.6	520.78	165.13	247.98	107.67	Current‐SSP1‐2.6	110.67	0.86	408.54
SSP2‐4.5	515.99	181.33	244.51	90.15	Current‐SSP2‐4.5	106.82	1.97	407.38
SSP3‐7.0	530.73	244.16	226.64	59.92	Current‐SSP3‐7.0	122.04	2.50	406.85
SSP5‐8.5	478.75	326.64	92.84	59.26	Current‐SSP5‐8.5	120.71	52.34	356.42

**TABLE 3:** The total potential distribution area of *N. mirabilis* in each country across different periods (/×10^4^ km^2^).

Country	LIG	LGM	MH	Current	SSP1‐2.6	SSP2‐4.5	SSP3‐7.0	SSP5‐8.5
Australia	1.34	0.00	0.00	20.82	41.93	45.29	49.93	44.20
Bangladesh	0.00	0.00	0.00	3.57	7.10	4.76	5.25	0.21
Bhutan	0.00	0.00	0.00	0.11	0.57	0.27	0.66	0.79
Brunei	0.00	0.39	0.45	0.45	0.45	0.45	0.45	0.04
Cambodia	0.00	0.00	0.17	8.10	11.61	12.06	9.69	5.69
China	0.00	0.00	0.00	40.01	63.55	65.81	66.08	73.40
Fiji	0.64	0.00	0.00	1.54	1.54	1.54	1.54	1.54
India	0.00	0.00	1.04	11.84	22.45	19.87	25.46	20.20
Indonesia	62.53	98.82	117.49	147.76	148.81	148.98	149.21	133.17
Laos	0.00	0.00	0.00	6.15	10.15	10.29	9.97	9.42
Malaysia	0.22	9.94	25.93	26.56	26.56	26.56	26.56	21.34
Myanmar	0.00	0.00	0.05	8.73	29.52	26.67	27.95	26.13
Nepal	0.00	0.00	0.00	0.01	3.80	1.94	3.59	4.95
New Caledonia	0.00	0.00	0.00	1.62	1.62	1.62	1.62	1.62
New Zealand	0.00	0.00	0.00	0.99	5.32	4.88	8.32	12.02
New Guinea	18.81	14.11	21.52	35.73	35.71	36.01	35.38	31.54
Philippines	0.07	4.33	8.76	23.56	23.78	23.61	23.68	19.02
Singapore	0.00	0.00	0.04	0.04	0.04	0.04	0.04	0.04
Solomon Islands	2.01	2.04	2.06	2.10	2.10	2.10	2.08	0.84
Sri Lanka	0.00	0.26	1.24	4.80	5.30	5.35	5.30	5.05
Thailand	0.00	0.00	2.93	6.49	9.94	9.55	8.85	7.99
Timor Leste	0.88	0.00	0.00	1.15	1.21	1.21	1.21	1.19
Vanuatu	0.72	0.00	0.00	0.93	0.93	0.93	0.93	0.93
Vietnam	0.00	0.00	0.01	21.04	25.95	26.21	26.18	25.03

During the LIG period, suitable habitats were mainly located in Indonesia (62.53 × 10^4^ km^2^), New Guinea (18.81 × 10^4^ km^2^), the Solomon Islands (2.01 × 10^4^ km^2^), and Australia (1.34 × 10^4^ km^2^). Between the LIG and LGM periods, the suitable area expanded by 89.63 × 10^4^ km^2^ (96.72%) and contracted by 48.76 × 10^4^ km^2^ (52.62%). During the LGM, the primary suitable areas shifted to Indonesia (98.82 × 10^4^ km^2^), New Guinea (14.41 × 10^4^ km^2^), Malaysia (9.94 × 10^4^ km^2^), and the Philippines (4.33 × 10^4^ km^2^). Notably, suitable areas previously present in Australia, Timor‐Leste, Fiji, and Vanuatu during the LIG had disappeared by the LGM. From the LGM to the MH period, the suitable area expanded by 62.44 × 10^4^ km^2^ (31.67%) and contracted by 57.98 × 10^4^ km^2^ (29.41%). During the MH, the main suitable areas were found in Indonesia (117.49 × 10^4^ km^2^), Malaysia (25.93 × 10^4^ km^2^), New Guinea (21.52 × 10^4^ km^2^), and the Philippines (8.76 × 10^4^ km^2^). The increase in suitable area in Malaysia exceeded that of New Guinea, making Malaysia the second largest distribution region for *N. mirabilis* during the MH. By this period, the suitable distribution continued expanding toward higher latitudes (Table S4), extending into Thailand, Vietnam, Cambodia, Myanmar, and Singapore (Table 3). Between the MH and the present, the distribution area expanded by 209.98 × 10^4^ km^2^ (104.16%) while contracting by only 3 × 10^4^ km^2^ (1.49%). Under current climatic conditions, the suitable distribution area of *N. mirabilis* spans 25 countries, with the largest areas found in Indonesia, China, New Guinea, and Malaysia. The expansion toward higher latitudes has continued (Table S4), with more high‐latitude countries now exhibiting suitable habitats for the species (Table 3).

The vegetation types of Southeast Asia during the LGM period, as illustrated in Figure S3, were predominantly composed of tropical rainforests, monsoon forests, tropical woodland, and tropical grasslands. When integrated with the potential suitable distribution of *N. mirabilis* during the LGM period, the results indicate that *N. mirabilis* primarily occurred in tropical rainforest and monsoon forest environments (Figures 4 and S3).

### 3.3 Prediction of Potential Distribution and Dynamic Distribution Under Different Future Scenarios

Under different future climate scenarios, the potential suitable distribution areas of *N. mirabilis* show distinct differences (Figure 4). Overall, compared to the current climate, the total suitable habitat area of *N. mirabilis* is projected to expand significantly under all four future climate scenarios.

We used four Shared Socioeconomic Pathways (SSPs), which represent different greenhouse gas emission trajectories and socioeconomic development patterns projected by the IPCC. SSP1‐2.6 represents a low‐emission scenario characterized by strong climate mitigation efforts and sustainable development, leading to limited global warming. SSP2‐4.5 is a moderate scenario assuming intermediate mitigation and adaptation strategies, resulting in moderate increases in greenhouse gas emissions. SSP3‐7.0 describes a high‐emission scenario associated with regional rivalry, slower economic growth, and limited climate action, leading to substantial warming. Finally, SSP5‐8.5 represents the most extreme scenario, with continued reliance on fossil fuels and minimal mitigation efforts, resulting in the highest levels of greenhouse gas emissions and severe climate change impacts. These scenarios, presented here in order of increasing severity, provide a comprehensive framework for evaluating the potential future distributional responses of *N. mirabilis* under contrasting climate futures.

**FIGURE 5:**
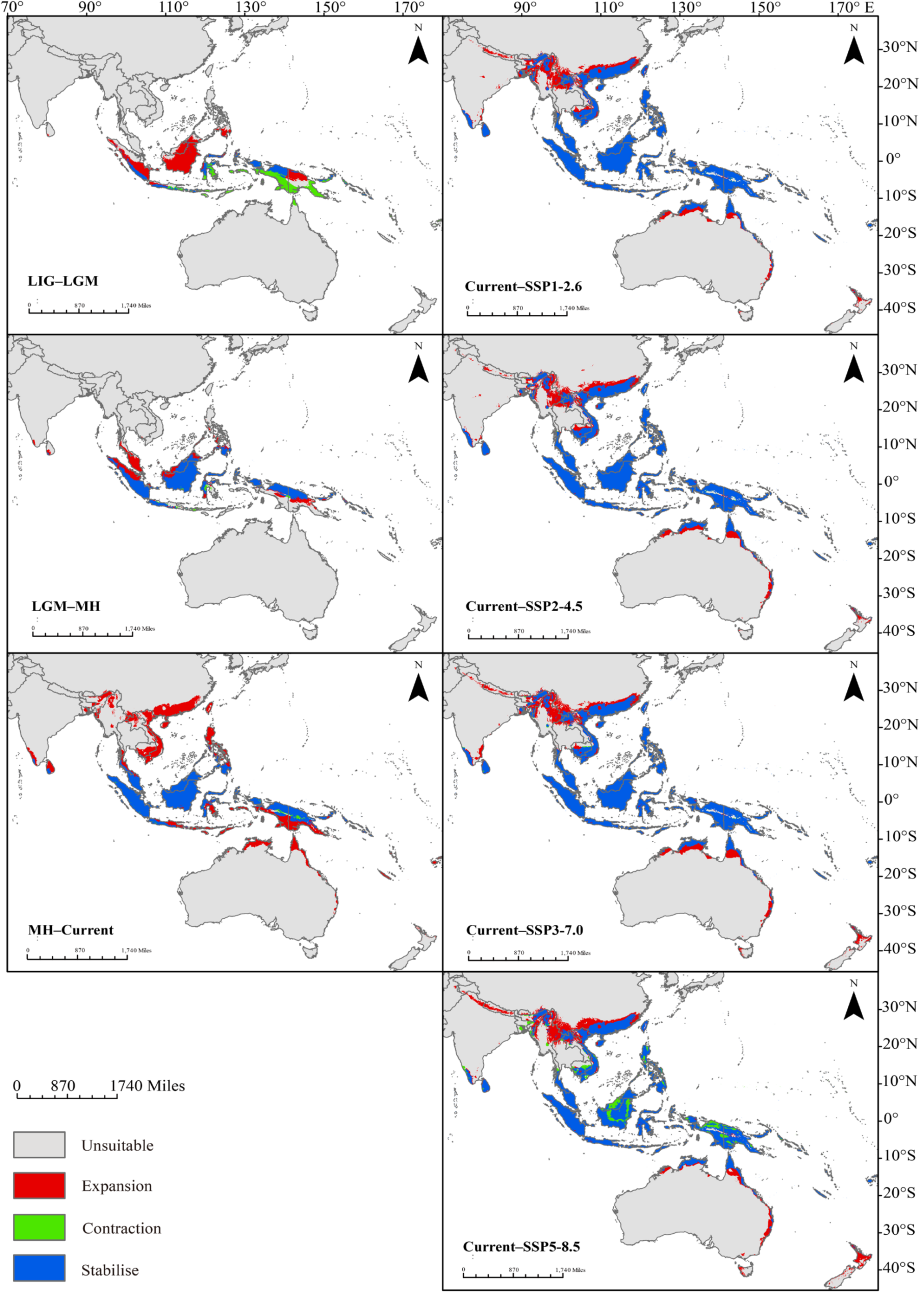
Changes in the potential suitable distribution of *Nepenthes mirabilis* under paleoclimatic, current, and future climate scenarios. Red areas and green areas represent range expansion and contraction, respectively; blue areas indicate stable habitats; and gray areas indicate unsuitable habitats.

The total suitable area is projected to be largest under the SSP3‐7.0 scenario, representing a 29.2% increase compared to the present climate (Table 2). However, in terms of high‐suitability habitats, *N. mirabilis* shows the greatest potential under the SSP1‐2.6 scenario, with a maximum high‐suitability area of 107.67 × 10^4^ km^2^ (Table 2). Meanwhile, as greenhouse gas emissions increase, the high‐suitability distribution area of *N. mirabilis* shows a decreasing trend. Under the SSP5‐8.5 scenario, the high‐suitability distribution area of *N. mirabilis* is 52.9% smaller than that under the SSP1‐2.6 scenario (Figure 4; Table 2). In contrast, the low‐suitability distribution area increases as greenhouse gas emissions rise (Figure 4; Table 2). Furthermore, under the SSP1‐2.6 and SSP2‐4.5 scenarios, the proportion of the moderate suitability distribution area relative to the total suitable distribution area is the largest, accounting for 47.6% and 47.4%, respectively. However, under the SSP3‐7.0 and SSP5‐8.5 scenarios, the proportion of the low‐suitability distribution area relative to the total suitable distribution area is the largest, at 46% and 68.2%, respectively (Table 2). Meanwhile, under all four scenarios, the expanded distribution area is significantly larger than the contracted area compared to the current climate. However, under the SSP5‐8.5 scenario, the suitable distribution area of *N. mirabilis* experiences a substantial loss (Figure 5; Table 2).

**FIGURE 6:**
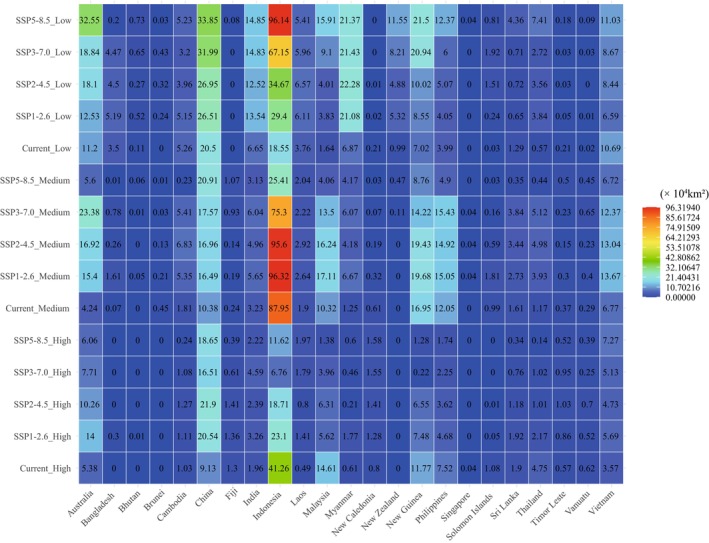
Heatmap showing the distribution area of *Nepenthes mirabilis* at different suitability levels across countries under current and future climate scenarios. Suitability is categorized into high, medium, and low based on logistic output thresholds. The color gradient from blue to red represents increasing habitat area.

An analysis of the potential changes in suitable distribution of *N. mirabilis* across various countries under different future scenarios reveals that low‐latitude countries such as Indonesia, Malaysia, and New Guinea experience a decline in high‐suitability distribution areas with the increase of greenhouse gas emission levels (Figure 6). For example, the high‐suitability areas in Indonesia are projected to decrease under all four future scenarios, with a maximum area of 23.1 × 10^4^ km^2^ under SSP1‐2.6, which is substantially lower than the current extent of 41.26 × 10^4^ km^2^. In contrast, relatively high‐latitude countries such as China, Australia, and New Zealand experience an expansion of high‐suitability distribution areas for *N. mirabilis* with the increase in greenhouse gas emission levels (Figure 6). For example, the high‐suitability areas in China are expected to expand under all four future scenarios, with a maximum projected area of 21.9 × 10^4^ km^2^ under SSP2‐4.5, substantially higher than that under current conditions (9.13 × 10^4^ km^2^).

### 3.4 Historical Migration Routes of Suitable Distribution Ranges for *N. mirabilis*

**FIGURE 7:**
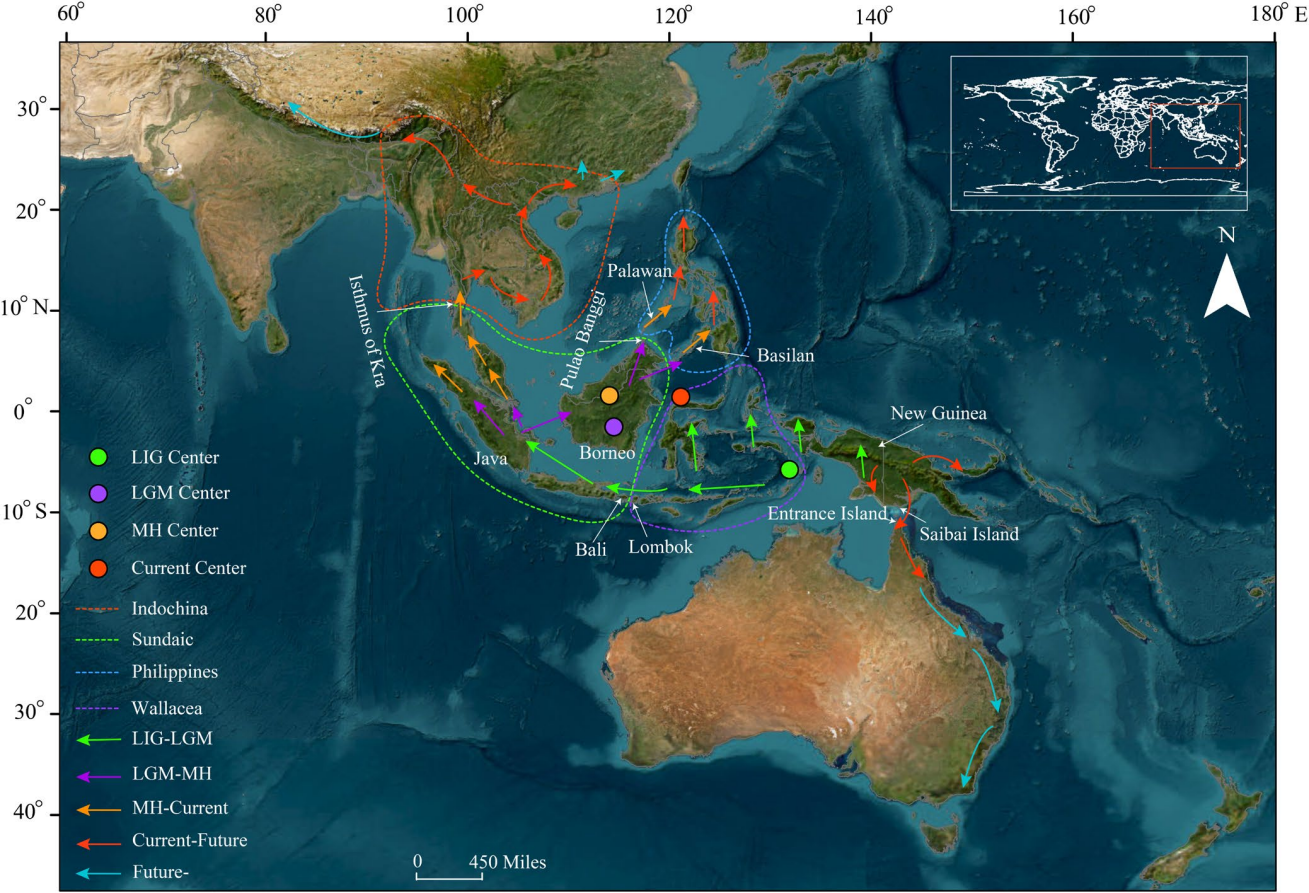
Centroid locations and inferred migration routes of *Nepenthes mirabilis* across different time periods. Green, purple, orange, and red solid circles represent the distribution centroid during the Last Interglacial (LIG), Last Glacial Maximum (LGM), Mid‐Holocene (MH), and current climate, respectively. Dashed lines delineate biogeographic subregions: Red for Indochina, green for Sundaic, blue for the Philippines, and purple for Wallacea. Arrows indicate the inferred migration routes: Green (from LIG to LGM), purple (LGM to MH), orange (MH to current), red (current to future), and blue (projected future scenarios under climate change).

During the LIG period, *N. mirabilis* was predicted to be primarily distributed in Wallacea, Sundaic, and New Guinea, the former two of which were connected via the Lombok and Bali islands (Figure 7). By the LGM period, the suitable distribution range of *N. mirabilis* shifted overall toward equatorial regions, with an expansion in the Sundaic region (Figures 4 and 7). By the MH period, the suitable distribution range of *N. mirabilis* expanded into the Philippines through Palawan from Sundaic and into Indochina through the Isthmus of Kra from Sundaic, with the center of the suitable distribution range shifting northward (Figure 7). By the current period, the suitable distribution range of *N. mirabilis* has expanded toward higher latitudes in both northern and southern directions. In northern regions, the expansion is evident in the continued northward spread from the Philippine biogeographic subregion, as well as from the Indochina biogeographic subregion into Thailand and Cambodia, then into Vietnam, and further into China and Laos. From Laos, the distribution continues to expand into Myanmar and extends northwestward (Figure 7). In southern regions, it expanded from New Guinea to Australia. By the 2090s, the suitable distribution areas of *N. mirabilis* are suggested for a further continuous expansion toward north and south, gradually moving away from the equator. The higher the greenhouse gas emissions scenario, the more pronounced the expansion will be (Figures 5 and 7).

## 4 Discussion

Ecological niche models (ENM) are essential tools for predicting the impacts of climate change on the potential distribution of plant species (Ngarega et al. 2022). In the current study, we employed the MaxEnt model to predict the potential distribution of *Nepenthes mirabilis* in Southeast Asia under paleo, current, and future climate scenarios. This study represents the application of a niche modeling approach to simulate the distribution changes of *N. mirabilis* in Southeast Asia across different time periods. Although *N. mirabilis* was included in a previous ENM analysis, that study focused primarily on tropical regions in Southeast Asia and did not incorporate geographical records from China, resulting in an incomplete representation of the species' distribution (Schwallier et al. 2016). The two evaluation metrics of MaxEnt, AUC and TSS, consistently indicate the excellent performance of the distribution model for *N. mirabilis* , comparable to results from other studies based on *Nepenthes species* in Southeast Asia using the MaxEnt modeling approach (Renjana et al. 2024).

### 4.1 Suitable Environmental Conditions for the Distribution of ***N. mirabilis***

Climate oscillations along with environmental conditions have a significant impact on plant growth and reproduction, which are associated with growing habitat and geographical distribution range. The response to key environmental variables varies among different plant groups (Beaumont et al. 2005; Zhang et al. 2021; Layola et al. 2022). In this study, five key environmental variables, including Annual Mean Temperature, Temperature Seasonality, Annual Precipitation, April Precipitation, and December Precipitation, were all identified as influencing the distribution of *N. mirabilis* (Table 1). The response curves of the environmental variables indicate that *N. mirabilis* is suited to areas with high precipitation, meaning Annual Temperatures ranging from 22.11°C to 27.99°C and relatively stable ambient temperatures (Figure 3). Such environmental conditions are suggested to be linked to the geographical distribution pattern in lower‐latitude regions of *N. mirabilis* . Similar results were observed in *Betula*, where species inhabiting lower‐latitude regions in southwestern China tend to prefer more stable ambient temperatures than those distributed in higher‐latitude regions in northeastern China (Huang et al. 2025). Among all the five key environmental variables, Annual Precipitation is regarded as the most influential factor shaping the geographical distribution of *N. mirabilis* . Similar conditions were observed in other *Nepenthes* species, such as *N. tentaculata* and *N. khasiana* , whose suitable geographical distributions are also highly associated with Annual Precipitation (Gray et al. 2017; Konwar et al. 2023). Furthermore, based on the LGM vegetation map (Ray and Adams 2001), *N. mirabilis* was revealed to show distributions in vegetation types such as tropical rainforests or woodland, monsoon forests, and montane tropical forest, which are also considered to have served as glacial refugia for many plant species in Asia during the glacial period (Figures 4 and S3). Therefore, the most suitable environmental conditions for *N. mirabilis* are suggested to be warm and moist habitats, such as swamps, wetlands, or grasslands in low‐latitude regions based on the above evidence (Jebb and Cheek 1997; Clarke and Moran 2016).

The preference of *N. mirabilis* for high‐humidity and high‐temperature environments is intricately linked to its ecological habits (Bonhomme et al. 2011). As is known, *Nepenthes* plants normally grow in nutrient‐poor soils with low levels of nitrogen and phosphate. As it exclusively relies on photosynthesis, it is inadequate for their survival. Consequently, they have evolved special mechanisms to obtain the nitrogen they need, which depend on the predation of invertebrates to supply essential nitrogen resources (Schulze et al. 1999; Thornham et al. 2012). High‐humidity environments maintain moisture on the lips of the pitcher with slippery surfaces, facilitating the “slipping” of insects to fall into the trap, thus enhancing the likelihood of successful predation by *N. mirabilis* (Bohn and Federle 2004; Bauer et al. 2012).

### 4.2 Historical Origin and Migration Routes of *N. mirabilis*

Ecological niche modeling can help reveal the geographical distribution patterns of populations across different periods, providing insights into potential population dynamics when combined with empirical data for validation. Thus, we studied the population dynamic history and migration routes of *N. mirabilis* by analyzing its potentially suitable distribution range under paleoclimate and current climate, while acknowledging that empirical data would be required to further validate these model‐based inferences.

Southeast Asia is considered a region of high plant diversity with many species originating there (Bailey 1949; Buerki et al. 2014). Several ecologically and economically important plant species, such as *Colocasia esculenta* , *Musa acuminata* , *Myristica fragrans* , and *Saccharum officinarum* , trace their origins back to Southeast Asia, particularly within the Wallacean–New Guinea biogeographic zone (De Langhe 2009; Ahmed et al. 2020; Tripathi et al. 2016; Li et al. 2024; Li et al. 2025). In the current study, *N. mirabilis* was found to have been distributed mainly in regions of Wallacea and New Guinea during the LIG period (Figure 4; Table 3). Given the model‐inferred persistence of *N. mirabilis* in equatorial Southeast Asia across historical climatic fluctuations, it is plausible that low‐latitude regions near the equator—particularly the Wallacea and New Guinea regions—may have represented its ancestral distribution area (Figure 4). Our results show a strong concordance between phylogenomic and ENM analyses in inferring the origin of *N. mirabilis* . The phylogenomic study of Murphy et al. (2020), which included multiple populations of *N. mirabilis* across its distribution range, revealed that the earliest‐diverging lineages are located in Wallacea—particularly in Sulawesi (e.g., Gunung Lampia, Wawonii, and Morowali)—and in the western part of New Guinea. These phylogenetic patterns suggest that the ancestral range of *N. mirabilis* was likely centered in the Wallacean region and adjacent parts of New Guinea, from which subsequent westward and eastward dispersal events occurred. Consistently, our ENM results independently identified Wallacea and New Guinea as potential origin and refugial areas during the species' evolutionary history, reinforcing the idea that these regions played a pivotal role as centers of early diversification and dispersal for *N. mirabilis* . However, this inference should be interpreted with caution, not only because direct empirical or fossil evidence is lacking, but also because it assumes that the species' physiological tolerances have remained unchanged over time. Potential adaptive evolution, although not explicitly modeled here, should be considered as a caveat and represents an important direction for future research.

Regarding its population dynamics, the suitable habitat range of *N. mirabilis* did not contract during past climatic fluctuations but instead expanded continuously from the paleoclimate periods into the present (Figure 4; Tables 2 and 3). The expansion from the LIG to the LGM period can be attributed to the persistence of extensive tropical rainforests and savannas in the Sundaic region, which were suggested to be the glacial refugia for plant species in Southeast Asia (Heaney 1991; Raes et al. 2014; Figure S3). During the LGM period, the drop in sea level exposed the Sunda Shelf, forming land bridges facilitating plant migration. *Nepenthes mirabilis* was assumed to disperse from Wallacea to the Sundaic region via the land bridges of Lombok and Bali, and then from Java to Borneo across the exposed shelf, thus shifting its distribution center northwards to Borneo (Figure 7). These findings support the “Savanna Corridor” hypothesis proposed by Heaney (1991) and Ochoa et al. (2022). Compared to the LGM, the suitable distribution of *N. mirabilis* during the MH period expanded further northward into the Philippines and Indochina (Figures 4 and 7). This expansion likely occurred via Palawan and the Isthmus of Kra, respectively. However, the total suitable area during the MH did not increase significantly compared to that of the LGM (Table 2). This is probably due to the post‐glacial rise in global temperature, which caused glacier melting and sea‐level rise, submerging parts of the previously exposed Sundaic shelf and reducing available land for colonization. Additionally, extreme drought events recorded in stalagmite data from mainland Southeast Asia during the mid‐ to late‐Holocene are thought to have restricted the population expansion of *N. mirabilis* during the MH period (Griffiths et al. 2020). This highlights the key role of precipitation in shaping the geographical distribution of *N. mirabilis* , which is more adapted to warm and humid conditions. In the present day, the distribution of *N. mirabilis* has further expanded away from the equator, especially re‐entering the Australian continent along the eastern coastline from New Guinea and widely moving into the Philippines and Indochina (Figure 4). The formation of Philippine and Indochina biogeographic subregions is suggested to be associated with the present‐day vegetation types in Southeast Asia, driven by the humid tropical conditions maintained by the seasonal reversal of monsoon winds from East Asia and Australia (Gagan et al. 2004; Wurster et al. 2010).

Phylogeographic evidence also supports these findings that the ancestral range of *N. mirabilis* has been spreading northwards to the Philippines, Borneo, and other regions with higher latitudes based on molecular data (Biswal et al. 2018; Murphy et al. 2020). As is known, the most plausible explanation for the genus's disjunct distribution is long‐distance dispersal events prior to the rapid diversification of the genus within Malesia (Clarke et al. 2017). The ability of long‐distance dispersal in *Nepenthes* has also been hypothesized in other species such as *N. khasiana* (Biswal et al. 2018; Konwar et al. 2023). Long‐distance dispersal of *Nepenthes* occurred primarily through wind, thanks to their long, wing‐like appendages. Other dispersal methods include water (seeds can float) and animals, although this is less common. Humans also contribute to seed dispersal over long distances (Clarke et al. 2017). Overall, our findings suggest that *N. mirabilis* probably originated in Wallacea and New Guinea, and achieved widespread distribution across Southeast Asia through a combination of land bridge‐mediated long dispersal during glacial periods and climatic facilitation during interglacials. Geological and climatic changes in Southeast Asia from the paleoclimate periods to the present not only provided favorable conditions for the growth and reproduction of *N. mirabilis* , but also facilitated its diffusion. The species' ecological flexibility and dispersal capacity have played essential roles in shaping their current distribution.

### 4.3 Response to Future Environmental Challenges and Conservation Strategies for *N. mirabilis*

In our study, the Sustainable Development Pathway (SSP1‐2.6) is identified as the most favorable future climate scenario for the persistence of *N. mirabilis* . Although the largest total suitable area is projected under the SSP3‐7.0 scenario, the extent of high‐suitability habitat is greatest under SSP1–2.6, reaching a maximum of 107.67 × 10^4^ km^2^ and accounting for 20.67% of the total area, compared to only 11.30% under SSP3‐7.0 (Table 2). Therefore, we propose that the SSP1‐2.6 scenario provides the most suitable climatic conditions for the future survival and development of *N. mirabilis* .

Notably, under all four future climate scenarios, the potential suitable distribution range of *N. mirabilis* is projected to expand, with expansion areas exceeding contraction areas in all cases (Figure 5; Tables 2 and S4). Climate warming is likely facilitating the expansion of *N. mirabilis* , similar to its role in promoting the range expansion of plant species in the Himalaya Hengduan Mountains (Liang et al. 2018; He et al. 2019). However, the response of *N. mirabilis* to climate warming under future scenarios varies by latitude. As is seen in the high‐latitude regions, a positive correlation with greenhouse gas emissions is shown (Figure 6; Table 2). Particularly, both China and Australia show a clear increase in suitable and high‐suitability distribution areas under future climate scenarios. In contrast, in low‐latitude regions, the suitable distribution area of *N. mirabilis* is somewhat negatively correlated with greenhouse gas emissions, with the high‐suitability areas being especially vulnerable to decline. Notably, under the SSP5‐8.5 scenario (General Development Pathway), low‐latitude countries face severe threats to the species' suitable distribution range. Despite an overall expansion trend, substantial losses of suitable habitats are projected in regions such as Indonesia and Malaysia (Figure 5; Table 2). Similarly, areas of high suitability for *N. mirabilis* are expected to decline with increasing greenhouse gas emissions, particularly in low‐latitude areas, including the Wallacean–New Guinea region, Philippines, and Malaysia et al. (Figure 6).

The losses and contraction of both suitable and high‐suitability habitats in low‐latitude regions are mainly attributed to climate warming causing temperatures to exceed the maximum threshold of the Annual Mean Temperature (between 22.11°C and 27.99°C) for the optimal distribution of *N. mirabilis*, thus altering the habitat environment and reducing its suitability, potentially leading to the loss of suitable distribution areas under extreme climatic conditions. Additionally, human activities are considered one of the factors threatening the survival of *Nepenthes* species. In many parts of Southeast Asia, tropical forests have suffered from human‐induced destruction, and illegal commercial exploitation of *Nepenthes* species has led to a continuous decline in their populations (Gray et al. 2017; Renjana et al. 2024).

As climate change modifies the environment of current habitats, *N. mirabilis* in low‐latitude regions may be forced to seek new environments similar to its previous habitats for survival. In response to climate warming, model projections indicate a clear trend of potential range shifts toward higher latitudes in both the Northern and Southern Hemispheres (Figure 7). This is also the case in *Taxus* and wild soybeans, both of which shifted their distribution ranges to higher latitudes in the Northern Hemisphere under future warming scenarios (Poudel et al. 2014; He et al. 2016). Consequently, the future conservation and sustainable management of *N. mirabilis* require urgent actions, including substantial reductions in greenhouse gas emissions, a commitment to sustainable development, and efforts to mitigate global temperature rise. Additionally, protecting tropical rainforests, maintaining wetland habitats essential for *N. mirabilis* , and prohibiting the illegal commercial exploitation of its resources are crucial for its survival. To translate conservation goals into practical actions, both in situ and *ex situ* strategies should be implemented in a complementary manner. In situ conservation efforts should prioritize key refuge areas and biodiversity hotspots with stable climates, particularly high‐suitability zones identified in this study. Establishing protected areas, ecological corridors, and habitat restoration programs in these zones can help maintain natural populations. For example, based on the predicted distribution patterns under current and future climate scenarios, we recommend prioritizing high‐suitability habitats in northern Borneo and western New Guinea for in situ protection, as these regions are likely to serve as stable refugia and maintain long‐term population viability. Conservation actions in these areas should focus on preserving existing natural habitats, controlling land‐use change, and mitigating deforestation pressures. *Ex situ* conservation efforts, including seed banking, botanical gardens cultivation, and nursery propagation, should target genetically diverse populations from both core and edge regions. By linking these strategies directly to the spatial distribution patterns identified in our modeling, we provide practical guidance for conserving *N. mirabilis* in the face of ongoing climate change. These strategies serve as insurance against future habitat loss and provide material for potential reintroduction or assisted migration if natural populations decline. Together, these integrated strategies will provide a practical framework for mitigating extinction risk and supporting the resilience of *N. mirabilis* populations under climate change.

## 5 Conclusion

In this study, we utilized the MaxEnt model to simulate the suitable distribution of *N. mirabilis* under past, present, and future climatic conditions, predict the species' origin and migration routes, and assess the impact of climate change on its future development. The results indicate that climate change significantly affects the suitable distribution of *N. mirabilis* . Annual precipitation is identified as the most important environmental variable for *N. mirabilis* , which is suggested to best thrive in warm and humid environments. The model predicts that *N. mirabilis* likely originated in Wallacea and New Guinea, followed by expansion and migration from low latitude to high latitude away from the equator, in response to climate change. The unique geographical features of Southeast Asia's islands are suggested to act as stepping stones, spreading from Wallacea to the Sundaic, Philippines, and Indochina during the colonization of *N. mirabilis* . Under future global warming scenarios, the Sustainable Development Pathway is identified as the most favorable future climate scenario for the persistence of *N. mirabilis* . With the rising temperature in the future, *N. mirabilis* is expected to continue migrating and expanding toward higher latitudes, with the trend becoming more pronounced as greenhouse gas emission levels increase. However, the suitable and high‐suitability distribution ranges contracted and the survival of *N. mirabilis* seems to be threatened in low‐latitude regions due to climate warming. Ecological niche modeling of *N. mirabilis* across different time periods explores its dynamic history and future development, revealing the species' geographical origin and migration routes. Both in situ and *ex situ* conservation strategies are proposed to safeguard the wild germplasm resources of *N. mirabilis* and promote its sustainable development, thereby enhancing the species' capacity to cope with future climate change. This study not only provides strong evidence for the study of other vegetation types in Southeast Asia but also offers new insights into the proper conservation of *N. mirabilis* populations.

## Author Contributions

**Wei Gong:** conceptualization (equal), methodology (equal), project administration (equal), supervision (equal), writing – review and editing (equal). **Hanghui Kong:** conceptualization (equal), methodology (equal), supervision (equal), writing – review and editing (equal). **Zhilong Huang:** data curation (equal), formal analysis (equal), writing – original draft (equal). **Wenya Yu:** data curation (equal), writing – review and editing (equal). **Wenjun Lv:** data curation (equal), writing – review and editing (equal). **Chuxin Liang:** data curation (equal), writing – review and editing (equal). **Rongjing Zhang:** resources (equal), writing – review and editing (equal). **Archie Along:** resources (equal).

## Funding

This work was supported by the National Natural Science Foundation of China (Grant 32270218; 32370240).

## Conflicts of Interest

The authors declare no conflicts of interest.

## Supporting information


**Figure S1:** Pearson correlation coefficients and Variance Inflation Factor (VIF) values for the environmental variables. Variables showing high collinearity (|*r*| > 0.7) or strong multicollinearity (VIF > 2.5) were excluded from the final MaxEnt model.

**Figure S2:** Evaluation metrics used for MaxEnt model parameter optimization. (a) Difference in corrected Akaike Information Criterion (Delta.AICc); (b) Difference between training and test AUC values (AUC.DIFF); (c) 10% training omission rate (OR10). RM indicates the regularization multiplier.

**Figure S3:** Global vegetation distribution during the Last Glacial Maximum (LGM) (modified from Ray and Adams 2001). Suitable habitats of Nepenthes mirabilis during the LGM period were predominantly distributed within vegetation types classified as 1 – Tropical rainforest, 2 – Monsoon or dry forest, and 10 – Montane tropical forest, particularly across the Wallacea and New Guinea regions of Southeast Asia.

**Appendix S1:** ece372707‐sup‐0005‐AppendixS1.docx.

**Video S1:** ece372707‐sup‐0004‐VideoS1.mp4.

## Data Availability

The species distribution records utilized in this study were obtained from the Global Biodiversity Information Facility (GBIF) and the Chinese Virtual Herbarium (CVH). Climatic variables were sourced from the WorldClim database. All geographic distribution data and associated environmental variables have been archived and are publicly accessible via the following Figshare repository: https://figshare.com/articles/dataset/Ecology_and_Evolution_‐_Manuscript_ID_ECE‐2025‐07‐01906_zip/29631905.
