# Supplementary figures and images for: Ecological Niche Modeling Reveals Historical Population Dynamics and Future Climate Response of the Carnivorous Plant Nepenthes mirabilis in Southeast Asia
Source: Ecol Evol. 2025 Dec 16;15(12):e72707. doi: 10.1002/ece3.72707 (PMC12706176; doi:10.1002/ece3.72707)

**a.**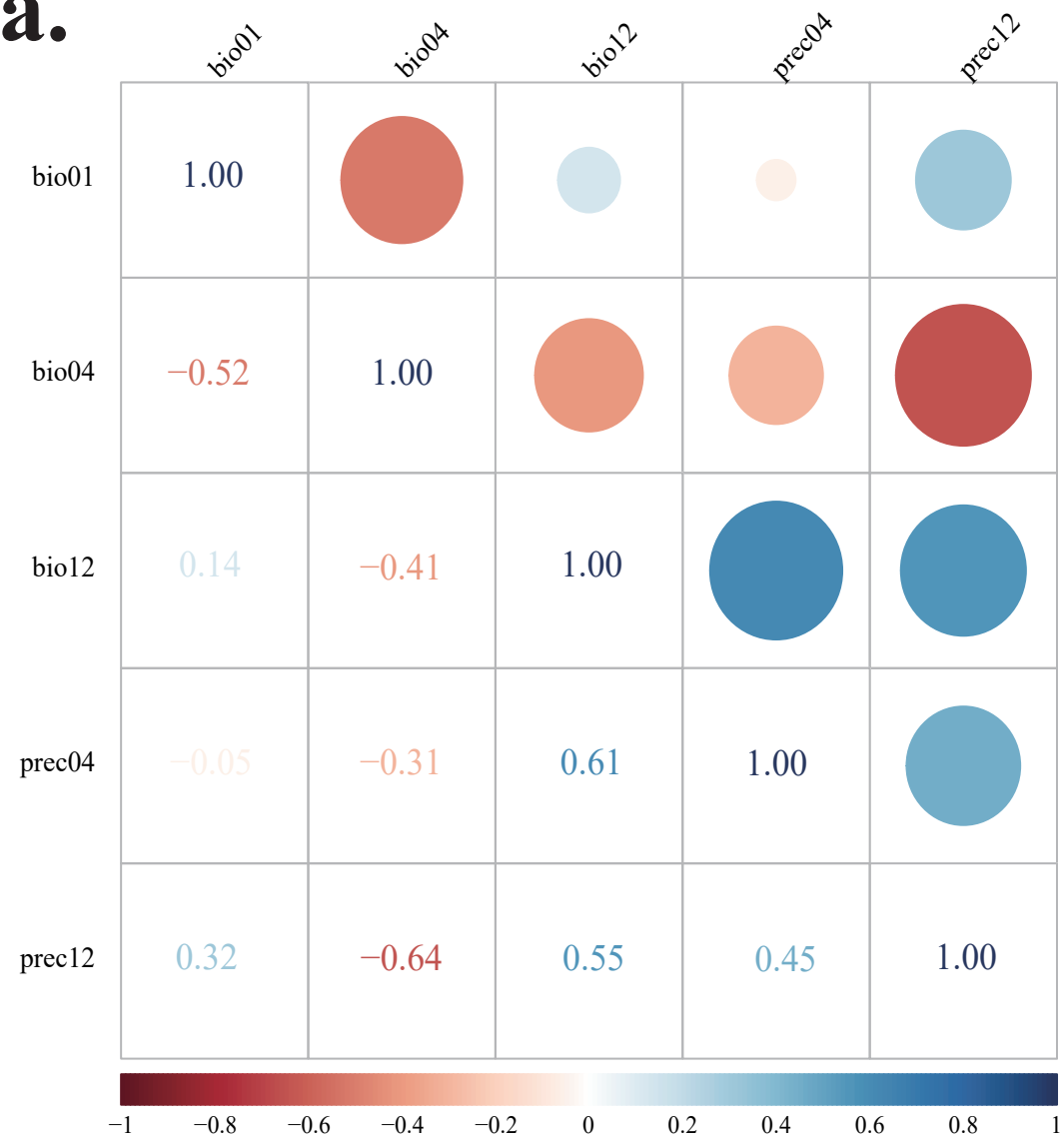**b.**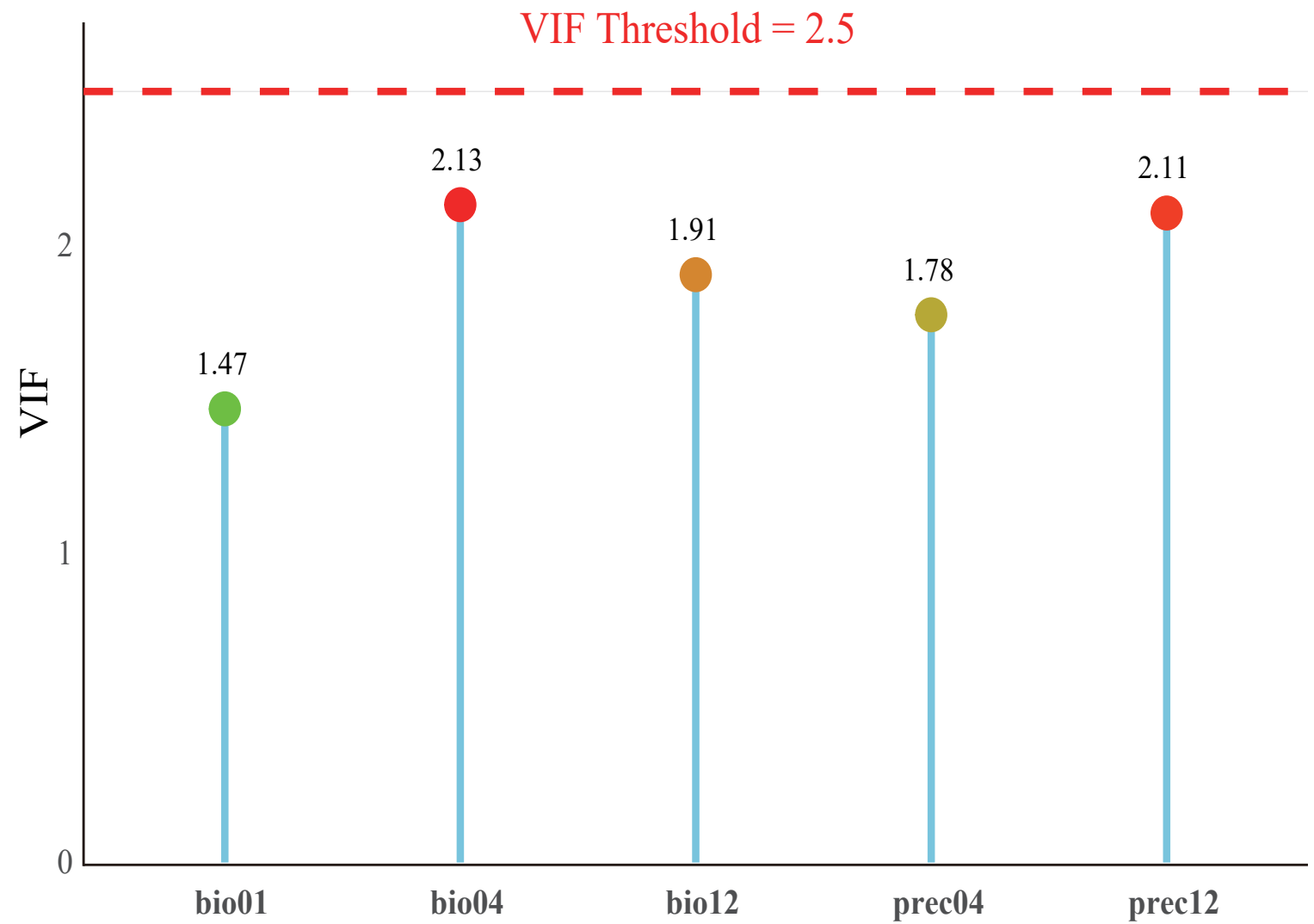

Supplement: Supplementary file 1 — Figure S1: Pearson correlation coefficients and Variance Inflation Factor (VIF) values for the environmental variables. Variables showing high collinearity (|r| > 0.7) or strong multicollinearity (VIF > 2.5) were excluded from the final MaxEnt model. [file ECE3-15-e72707-s004.pdf]

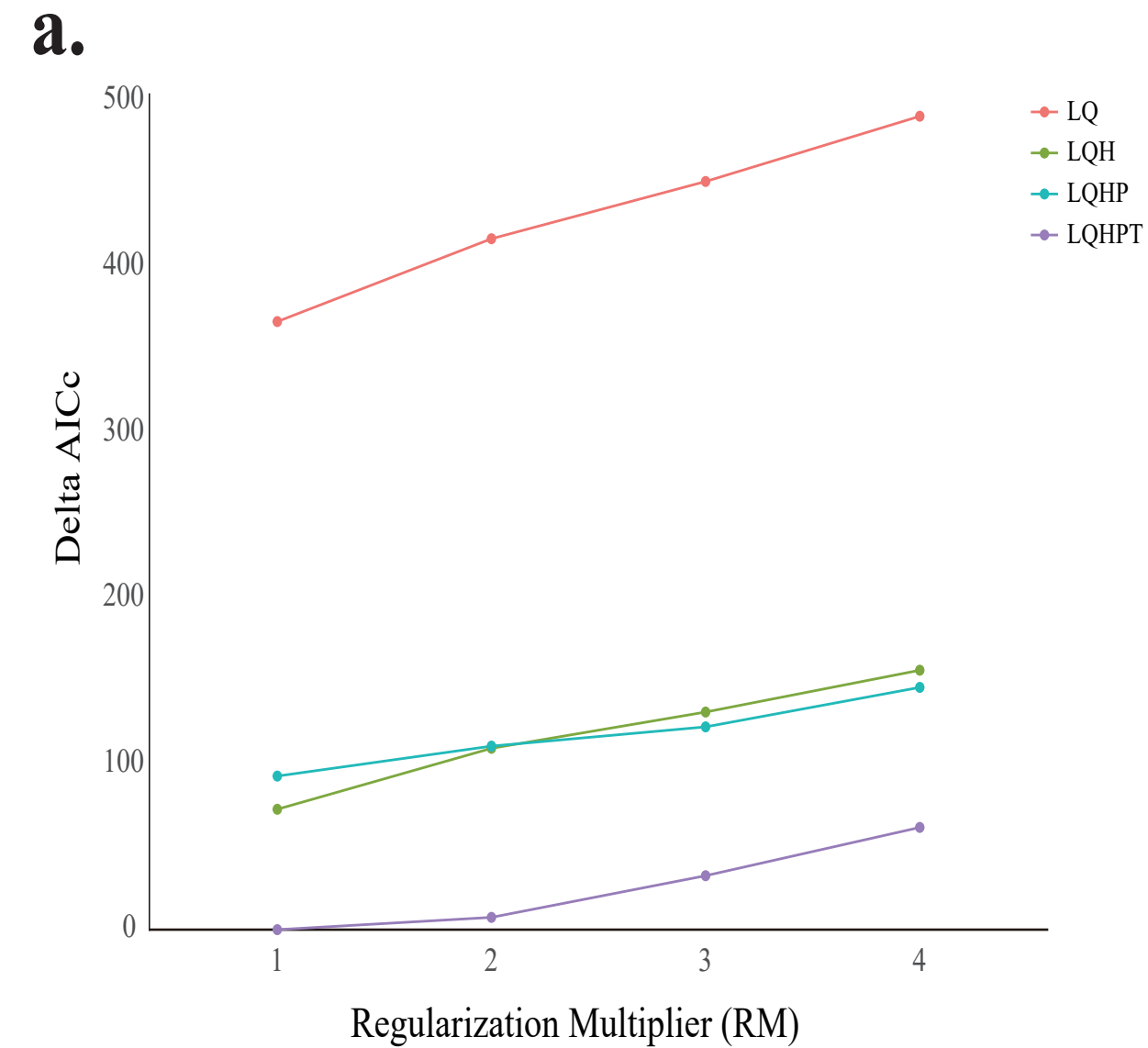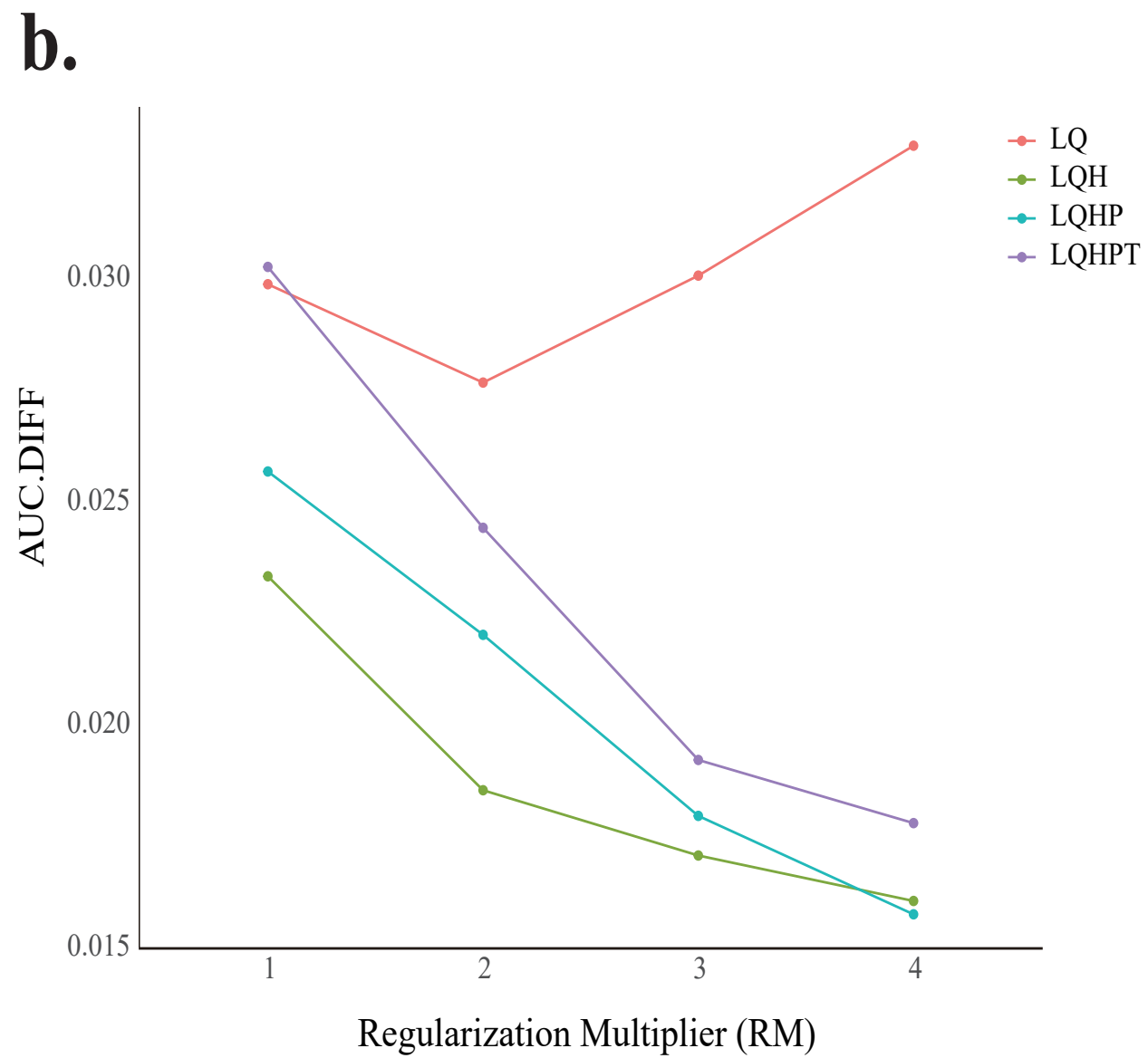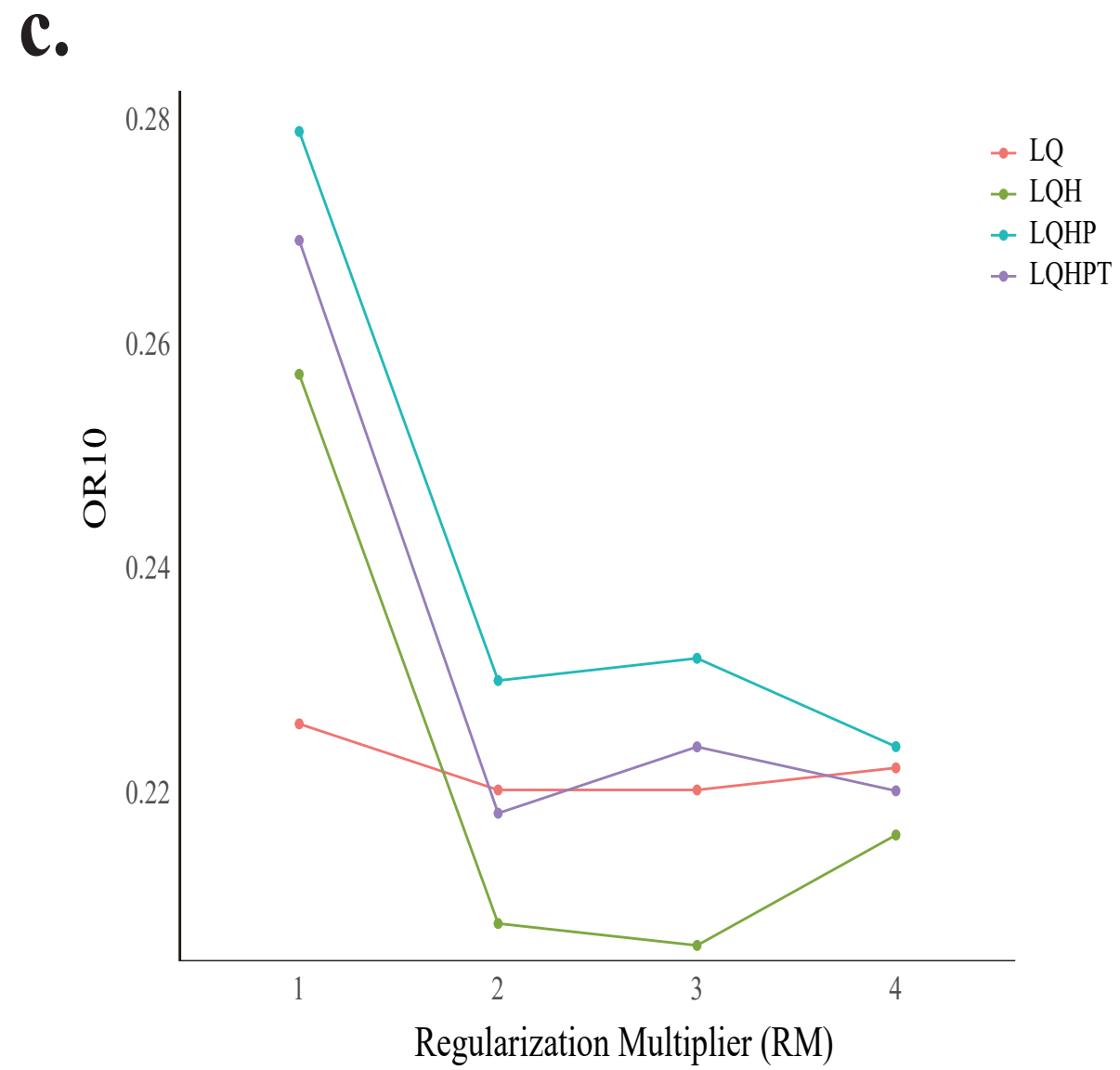

Supplement: Supplementary file 2 — Figure S2: Evaluation metrics used for MaxEnt model parameter optimization. (a) Difference in corrected Akaike Information Criterion (Delta.AICc); (b) Difference between training and test AUC values (AUC.DIFF); (c) 10% training omission rate (OR10). RM indicates the regularization multiplier. [file ECE3-15-e72707-s005.pdf]
