# Supplementary material for: Ecological Niche Modeling Reveals Historical Population Dynamics and Future Climate Response of the Carnivorous Plant Nepenthes mirabilis in Southeast Asia
Source: Ecol Evol. 2025 Dec 16;15(12):e72707. doi: 10.1002/ece3.72707 (PMC12706176; doi:10.1002/ece3.72707)
Supplement: Supplementary file 3 — Figure S3: Global vegetation distribution during the Last Glacial Maximum (LGM) (modified from Ray and Adams 2001). Suitable habitats of Nepenthes mirabilis during the LGM period were predominantly distributed within vegetation types classified as 1 – Tropical rainforest, 2 – Monsoon or dry forest, and 10 – Montane tropical forest, particularly across the Wallacea and New Guinea regions of Southeast Asia. [file ECE3-15-e72707-s003.pdf]

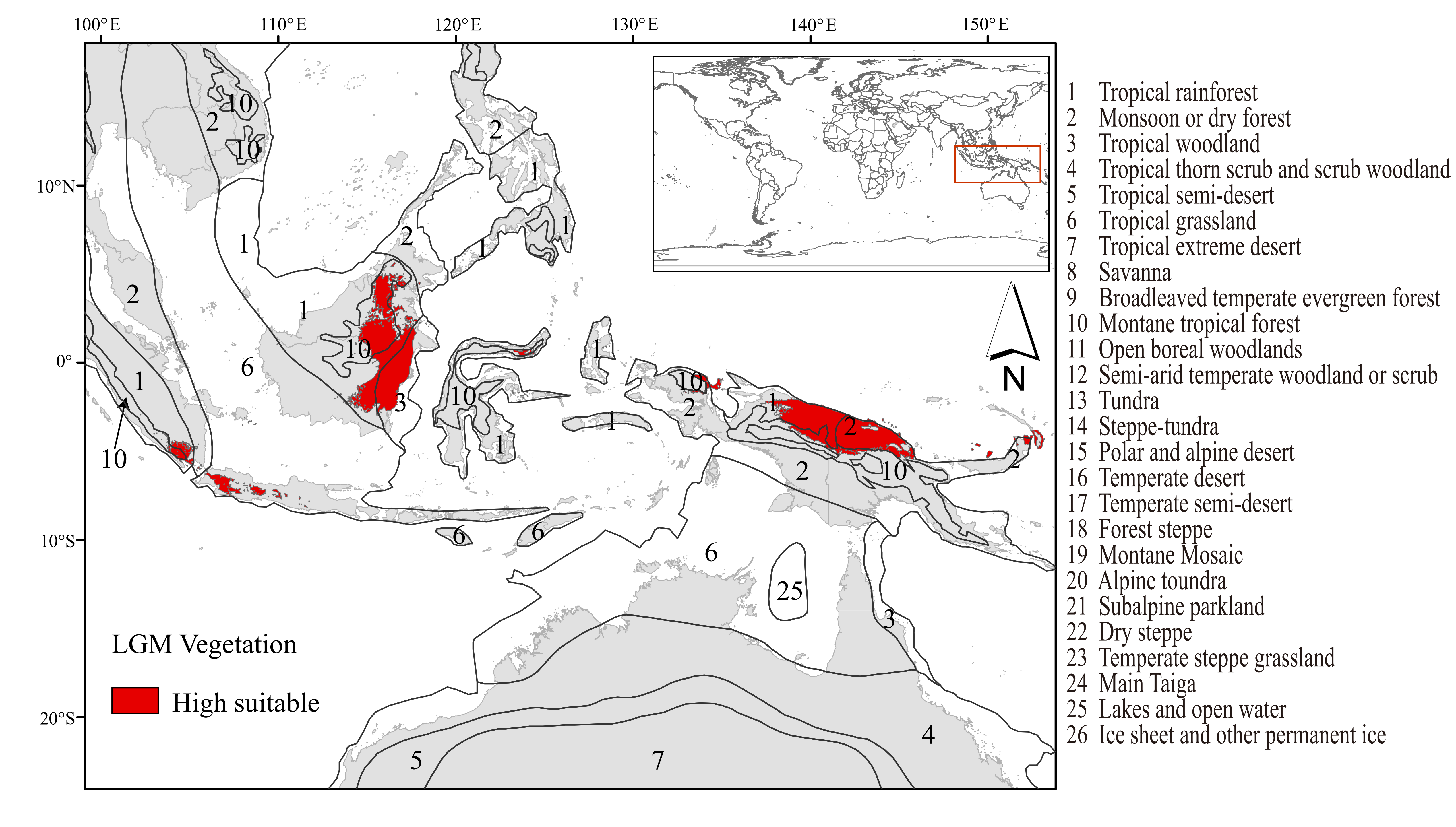
100°E
110°E
120°E
130°E
140°E
150°E
10°N
0°
10°S
20°S
LGM Vegetation
High suitable
N
1 Tropical rainforest
2 Monsoon or dry forest
3 Tropical woodland
4 Tropical thorn scrub and scrub woodland
5 Tropical semi-desert
6 Tropical grassland
7 Tropical extreme desert
8 Savanna
9 Broadleaved temperate evergreen forest
10 Montane tropical forest
11 Open boreal woodlands
12 Semi-arid temperate woodland or scrub
13 Tundra
14 Steppe-tundra
15 Polar and alpine desert
16 Temperate desert
17 Temperate semi-desert
18 Forest steppe
19 Montane Mosaic
20 Alpine toundra
21 Subalpine parkland
22 Dry steppe
23 Temperate steppe grassland
24 Main Taiga
25 Lakes and open water
26 Ice sheet and other permanent ice
