# Supplementary material for: Ecological Niche Modeling Reveals Historical Population Dynamics and Future Climate Response of the Carnivorous Plant Nepenthes mirabilis in Southeast Asia
Source: Ecol Evol. 2025 Dec 16;15(12):e72707. doi: 10.1002/ece3.72707 (PMC12706176; doi:10.1002/ece3.72707)
Supplement: Supplementary file 4 — Appendix S1: ece372707‐sup‐0005‐AppendixS1.docx. [file ECE3-15-e72707-s002.docx]

**Supplementary Table**

**Figure Legends**

**Fig. S1. Pearson correlation coefficients and Variance Inflation Factor (VIF) values for the environmental variables.** Variables showing high collinearity (|r| > 0.7) or strong multicollinearity (VIF > 2.5) were excluded from the final MaxEnt model.

**Fig. S2. Evaluation metrics used for MaxEnt model parameter optimization.** (a) Difference in corrected Akaike Information Criterion (Delta.AICc); (b) Difference between training and test AUC values (AUC.DIFF); (c) 10% training omission rate (OR10). RM indicates the regularization multiplier.

**Fig. S3. Global vegetation distribution during the Last Glacial Maximum (LGM) (modified from Ray and Adams, 2001).** Suitable habitats of *Nepenthes mirabilis* during the LGM period were predominantly distributed within vegetation types classified as 1 – Tropical rainforest, 2 – Monsoon or dry forest, and 10 – Montane tropical forest, particularly across the Wallacea and New Guinea regions of Southeast Asia.

**Tables**

Table S1 Initial environmental variable contribution and importance values.

| Variable | contribution | importance |
| --- | --- | --- |
| bio12 | 21 | 1 |
| prec04 | 18.3 | 6.2 |
| bio04 | 6.4 | 6.2 |
| bio06 | 4.7 | 0.2 |
| bio07 | 3.9 | 0.6 |
| tmin10 | 3.2 | 1.4 |
| prec12 | 3.1 | 6.8 |
| bio14 | 2.9 | 1.4 |
| prec02 | 2.5 | 4.5 |
| tmin07 | 2.3 | 0.4 |
| bio11 | 2.3 | 0 |
| bio03 | 2.2 | 2.8 |
| bio01 | 1.9 | 0.2 |
| slopescl6 | 1.9 | 1.4 |
| slopescl1 | 1.8 | 0.8 |
| tmin08 | 1.4 | 0.2 |
| bio15 | 1.2 | 2.6 |
| tmin09 | 1.2 | 0.6 |
| slopescl2 | 1.2 | 2 |
| prec11 | 1.1 | 7.5 |
| prec09 | 0.8 | 3.9 |
| prec01 | 0.8 | 10.7 |
| slopescl8 | 0.6 | 1.4 |
| tmin12 | 0.6 | 0 |
| tmin11 | 0.6 | 0.5 |
| prec10 | 0.6 | 2.1 |
| tmax09 | 0.6 | 0.2 |
| prec03 | 0.5 | 1.9 |
| slopescl7 | 0.5 | 1.7 |
| aspectcln | 0.5 | 0.6 |
| slopescl5 | 0.5 | 0.5 |
| tmax10 | 0.5 | 0.1 |
| tmax11 | 0.5 | 0.2 |
| aspectclw | 0.5 | 0.5 |
| aspectcle | 0.5 | 1 |
| tmax01 | 0.4 | 0.8 |
| slopescl3 | 0.4 | 0.7 |
| tmin02 | 0.4 | 0.6 |
| bio17 | 0.4 | 4 |
| bio19 | 0.4 | 1.2 |
| elev | 0.4 | 4.6 |
| bio18 | 0.3 | 0.6 |
| tmin01 | 0.3 | 3.8 |
| tmin04 | 0.3 | 0 |
| aspectcls | 0.3 | 0.5 |
| prec07 | 0.3 | 1.4 |
| tmax06 | 0.3 | 0 |
| tmin05 | 0.3 | 1.3 |
| tmin03 | 0.3 | 2.2 |
| prec08 | 0.3 | 1 |
| prec05 | 0.3 | 1.2 |
| prec06 | 0.2 | 0.6 |
| bio10 | 0.2 | 0 |
| tmax04 | 0.2 | 0 |
| tmax07 | 0.2 | 0 |
| slopescl4 | 0.2 | 0.5 |
| tmax12 | 0.2 | 0.5 |
| tmax08 | 0.2 | 0 |
| tmax02 | 0.1 | 0.1 |
| bio05 | 0.1 | 0 |
| bio02 | 0.1 | 0.7 |
| tmax03 | 0.1 | 0 |
| bio09 | 0.1 | 0 |
| bio16 | 0.1 | 0.9 |
| bio08 | 0.1 | 0.3 |
| tmin06 | 0 | 0 |
| tmax05 | 0 | 0.3 |
| bio13 | 0 | 0.1 |

Table S2 Modulation multiplicity and four feature combinations.

| Feature combinations | Regularization multipliers |
| --- | --- |
| LQ | 1 |
| LQH | 1 |
| LQHP | 1 |
| LQHPT | 1 |
| LQ | 2 |
| LQH | 2 |
| LQHP | 2 |
| LQHPT | 2 |
| LQ | 3 |
| LQH | 3 |
| LQHP | 3 |
| LQHPT | 3 |
| LQ | 4 |
| LQH | 4 |
| LQHP | 4 |
| LQHPT | 4 |

Table S3 AUC and TSS values for each run and the mean from the Maxent model.

| Replications | AUC | TSS |
| --- | --- | --- |
| 1 | 0.9532 | 0.9532 |
| 2 | 0.9497 | 0.9418 |
| 3 | 0.9539 | 0.9303 |
| 4 | 0.9527 | 0.9527 |
| 5 | 0.9487 | 0.9487 |
| 6 | 0.9524 | 0.9524 |
| 7 | 0.9511 | 0.9432 |
| 8 | 0.9538 | 0.9459 |
| 9 | 0.9511 | 0.9432 |
| 10 | 0.9506 | 0.9427 |
| 11 | 0.9483 | 0.9404 |
| 12 | 0.9525 | 0.9525 |
| 13 | 0.9561 | 0.9561 |
| 14 | 0.9522 | 0.9522 |
| 15 | 0.9538 | 0.9538 |
| 16 | 0.9509 | 0.943 |
| 17 | 0.9506 | 0.9506 |
| 18 | 0.9563 | 0.9563 |
| 19 | 0.9516 | 0.9516 |
| 20 | 0.9542 | 0.9542 |
| Mean | 0.9522 | 0.9483 |

Table S4 Latitude and longitude ranges of suitable distribution areas for each period.

| Period | Lon. _max | Lon. _min | Lat. _max | Lat. _min |
| --- | --- | --- | --- | --- |
| Current | 179°56' E | 71°13' E | 34°31' N | 45°45' S |
| MH | 179°51' E | 71°18' E | 15°29' N | 15°41' S |
| LGM | 179°53' E | 71°7' E | 15°1' N | 15°41' S |
| LIG | 179°57' E | 96°48' E | 8°43' N | 19°33' S |
| SSP1-2.6 | 179°56' E | 71°13' E | 35°1'N | 48°46' S |
| SSP2-4.5 | 179°56' E | 71°13' E | 35°1'N | 48°46' S |
| SSP3-7.0 | 179°56' E | 71°13'E | 35°8'N | 49°56'S |
| SSP5-8.5 | 179°56' E | 71°13' E | 35°8' N | 49°56' S |

**Video**

**Video 1. Expansion and contraction of the suitable range of *Nepenthes mirabilis* from paleoclimatic to current climate.**
